# Leaf Phosphorus Fractions Are Coordinated with Leaf Functional Traits in Four Juvenile Tree Species from the Chinese Subtropics

**DOI:** 10.3390/plants14010004

**Published:** 2024-12-24

**Authors:** Lei Wang, Jinhong Guan, Zongpei Li, Zhijie Chen, Zaipeng Yu, Zhichao Xia

**Affiliations:** 1 Anhui Provincial Key Laboratory of Forest Resources and Silviculture, School of Forestry & Landscape Architecture, Anhui Agricultural University, Hefei 230036, China; wanglei3020@163.com (L.W.); lizongpei1999@163.com (Z.L.); 2 College of Life Sciences, Qinghai Normal University, Xining 810016, China; cocogjh@163.com; 3 Key Laboratory for Humid Subtropical Eco-Geographical Processes of the Ministry of Education, Institute of Geography, Fujian Normal University, Fuzhou 350117, China; chan.chijay@gmail.com (Z.C.); zaipengstephen@163.com (Z.Y.); 4 Fujian Provincial Key Laboratory for Subtropical Resources and Environment, School of Geographical Sciences, Fujian Normal University, Fuzhou 350117, China

**Keywords:** foliar P fractions, photosynthetic P-use efficiency, leaf functional traits, trade-off, soil properties, interspecific variation

## Abstract

Phosphorus (P) is essential for cellular processes, and P fractions within leaf tissues reflect distinct biochemical functions. However, the relationship among foliar P allocation, leaf functional traits, and soil properties in subtropical China remains poorly understood. Here, we investigated four tree species to examine the relationships among foliar P fractions (orthophosphate P, metabolic P, nucleic acid P, lipid P, and residual P), key leaf functional traits (LMA, A_mass_, and leaf [N], and P concentrations), and soil properties. A negative correlation between the relative allocation of metabolic P (rPM) and nucleic acid P (rPN) suggested a trade-off between metabolic activity and growth. LMA was positively correlated with rPM and residual P (rPR) but negatively associated with rPN and lipid P (rPL). Similarly, leaf [N] correlated positively with rPN and negatively with rPR. Structural equation modeling (SEM) revealed that foliar P allocation was primarily driven by leaf functional traits independent of soil properties. These findings underscore the critical role of leaf functional traits in shaping P allocation patterns and highlight the adaptive strategies of tree species to cope with P-deficient environments in subtropical ecosystems.

## 1. Introduction

Leaf phosphorus (P) is essential in plant physiology and is partitioned into five functional fractions, each reflecting distinct biochemical roles. Orthophosphate P (Pi) serves primarily as a storage form in vacuoles. Metabolite P (PM) includes low-molecular-weight compounds like ribulose bisphosphate (RuBP), nicotinamide adenine dinucleotide phosphate (NADP), adenosine triphosphate (ATP), and adenosine diphosphate (ADP), as well as storage compounds such as phytate. Nucleic acid P (PN) is found in ribonucleic acid (RNA) and deoxyribonucleic acid (DNA). Lipid P (PL) occurs mainly in phospholipids, especially in the membranes of the endoplasmic reticulum. Residual P (PR) comprises phosphorylated proteins and other unidentified P-containing compounds [1,2,3,4]. Concentrations and relative allocation of P among these fractions are intricately linked to various physiological processes within the leaf [1]. Specifically, excess P is often sequestered as Pi within vacuoles, facilitating P availability during periods of rapid growth or metabolic need [4,5]. In the PM fraction, key intermediates in photosynthetic and glycolytic pathways are included, reflecting the dynamic role of P in energy transformations and metabolic regulation [2]. Within the PN and PR fractions, their role is integral to the synthesis and turnover of nucleic acids and the phosphorylation of proteins, respectively, highlighting the fundamental role of P in genetic information processing and signal transduction [1,6,7]. Meanwhile, PL, which predominantly comprises phospholipids within cellular membranes, is critical for maintaining cellular integrity and facilitating intracellular communication [1].

Leaf functional traits, such as leaf mass per area (LMA), leaf lifespan (LL), and light-saturated net photosynthetic rate per unit dry mass (A_mass_), serve as bridges between plant ecological strategies and resource allocation patterns. Data from multiple species globally support the core tenets of the leaf economic spectrum (LES), indicating positive correlations between LL and LMA and negative correlations with A_mass_ [8,9]. For instance, the LES describes a spectrum of short-lived leaves with high A_mass_, indicating substantial resource investment in photosynthesis. These species also exhibit low LMA, which means they have fragile leaves. In contrast, long-lived leaves demonstrate lower A_mass_ and higher LMA, reflecting a greater resource investment in structural maintenance [10,11].

Several studies have explored the relationships among leaf P fractions, photosynthetic phosphorus-use efficiency (PPUE), which was defined as the ratio of the net photosynthetic rate to the leaf P content, and other key leaf functional traits within the framework of the LES [3,12,13]. Tsujii et al. [3] thoroughly analyzed how leaf P fractions were coordinated along the LES among 12 co-occurring Australian species. Tsujii et al. [12] also reported that species tended to exhibit lower concentrations of total foliar P, particularly in nucleic acids and storage P forms (Pi). In these species, a higher allocation of P to lipids (PL) was associated with reduced leaf [N], indicative of a resource-conserving strategy suited to nutrient-poor environments. This pattern aligns with the LES framework, where higher LMA and leaf [N] are typically linked to slower growth rates and more resource conservation. Gille et al. [13] further highlighted the variation in P allocation patterns in Banksia and Hakea species, which adopt highly conservative strategies. These species maintained high PPUE by allocating relatively less P to lipids and more to metabolites, which are critical for maintaining photosynthesis under nutrient constraints. Such findings provide critical insights into how variations in leaf P fractions, LMA, and leaf N concentrations ([N]) reflect species’ resource-use strategies across different ecological contexts.

Leaf P fractions exhibit considerable variability in response to soil properties [12,14,15,16,17]. As soil P availability decreases, total foliar P concentration also reduces, primarily due to reductions in metabolic and nucleic acid P. Concurrently, LMA increases, showing a shift toward more conservative resource-use strategies [14]. In a broader context, Liu et al. [18] highlighted that P allocation patterns among species from different families in southwestern Australia are highly species-dependent, underscoring the complexity of P allocation strategies across varied ecological contexts. Although soil N availability can significantly influence leaf P allocation through metabolic demands [19], the resulting effects are highly contingent upon species-specific traits and the prevailing ecological conditions [15,20]. For instance, N addition alone did not affect leaf P fractions in Chinese fir and *Salix paraplesia* but increased the rPM in *Abies fabri* [21,22]. In a long-term chronosequence study in Western Australia, *Acacia rostellifera* maintained a constant P-allocation pattern in its phyllodes, regardless of the transition from N to P limitation with increasing soil age, suggesting a stable P-allocation strategy. In contrast, *Hakea prostrata* reduced its P allocation to Pi, phospholipids, and nucleic acids as soil age increased. Meanwhile, *Melaleuca systena* exhibited the greatest variation in P allocation [15]. Plants modulate P allocation and utilization in accordance with their ecological strategies and resource availability. The interactions between the LES and soil properties likely play a crucial role in shaping leaf P fractions. However, it remains unclear how P allocation to specific fractions in young tree species, particularly under P-limited conditions, is linked to leaf functional traits.

P limitation poses a significant scientific challenge in understanding and managing tropical or subtropical forest ecosystems [23,24]. Our study explored the relationship between foliar P fractions and the LES and examined how leaf functional traits influenced leaf P fractions in relation to soil properties across four young tree species in southeastern China. Specifically, we tested two hypotheses: (1) Leaf functional traits were significantly correlated with leaf P fractions, such that leaf characteristics can predict the fractions of P within the leaves; (2) the impact of leaf functional traits on leaf P fractions is related to soil properties. Understanding these relationships is essential for elucidating plant growth strategies under environmental constraints, such as low soil fertility and P deficiency. Moreover, this investigation offers further insights into the application of the LES framework, advancing our understanding of how plants adapt to nutrient limitations.

## 2. Results

### 2.1. Variation in Leaf Functional Traits and Soil Properties

**Figure 1:**
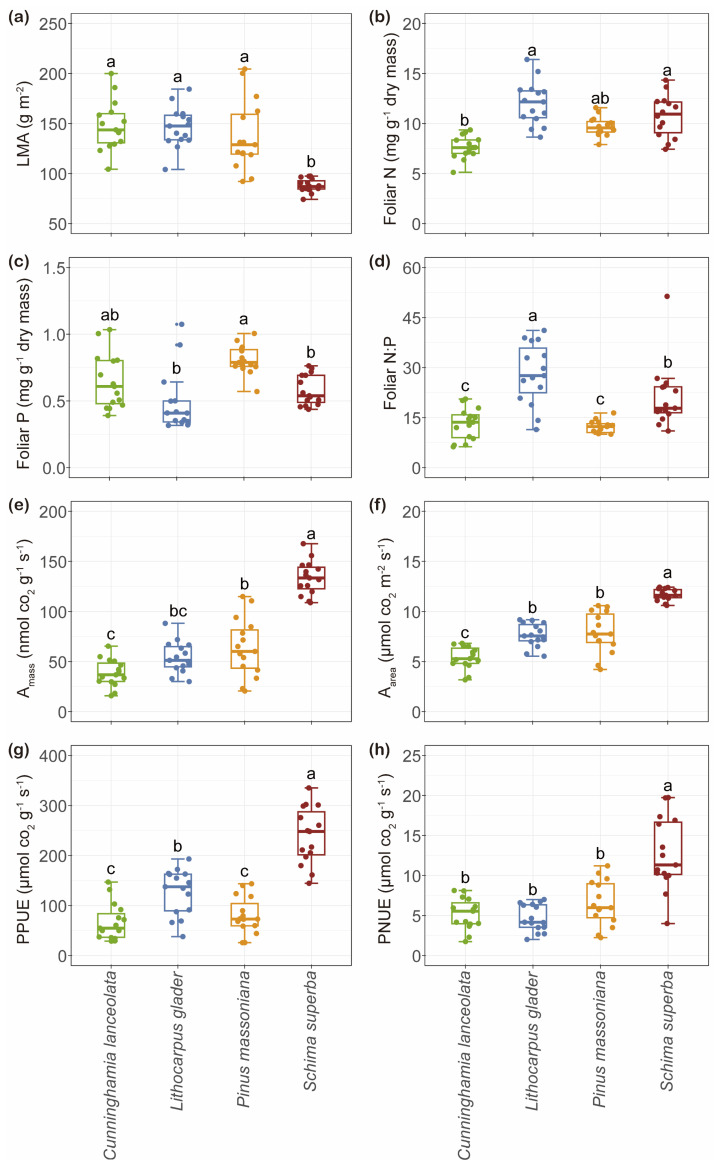
Box plots illustrate the variation in leaf economic traits among four tree species from the Chinese subtropics. The traits analyzed included leaf dry mass per unit area (LMA; (**a**)), leaf nitrogen concentration (foliar N; (**b**)), leaf phosphorus concentration (foliar P; (**c**)), nitrogen to phosphorus ratio (foliar N:P; (**d**)), light-saturated net photosynthetic rate per unit dry mass (A_mass_; (**e**)), light-saturated net photosynthetic rate per unit leaf area (A_area_; (**f**)), photosynthetic phosphorus-use efficiency (PPUE; (**g**)), and photosynthetic nitrogen-use efficiency (PNUE; (**h**)). Each box plot is color-coded to represent a different species, with each point on the plot representing an individual tree (*n* = 15). The central box of each plot displays the interquartile range and the median value for each species, while the whiskers extend to either 1.5 times the interquartile range or the most extreme data point. Statistical significance of pairwise differences among species is indicated by differing letters, based on Tukey’s Honest Significant Difference (HSD) test at a significance level of *p* < 0.05.

*Schima superba* exhibited a significantly lower LMA of 88.02 g m^−2^ compared to the other species, including *Cunninghamia lanceolata a* (148.08 g m^−2^), *Lithocarpus glaber* (146.36 g m^−2^), and *Pinus massoniana* (137.79 g m^−2^) (Figure 1a). In contrast, foliar N concentrations were similar among *L. glaber*, *P. massoniana*, and *S. superba* (12.01, 9.68, and 11.83 mg g⁻^1^, respectively), all of which were higher than that of *C. lanceolata* (Figure 1b). However, *P. massoniana* exhibited a higher foliar P concentration (0.80 mg g^−1^) than *L. glaber* and *S. superba* (0.48 and 0.58 mg g^−1^) (Figure 1c). Among the species, *L. glaber* had the highest N: P (28.18) ratio (Figure 1d). Notably, *S. superba* demonstrated the highest A_mass_, A_area_, PPUE, and PNUE (Figure 1e–h).

The physicochemical properties of soils under four distinct tree species exhibited no significant variance in terms of pH, soil water content (SWC), total nitrogen (TN), total carbon (TC), nitrate nitrogen (NO_3_^−^-N), and ammonium nitrogen (NH_4_^+^-N) concentrations (Table S1). Notably, soil total phosphorus (TP) under *S. superba* (6.70 mg kg^−1^) growth was significantly lower compared to those found under *C. lanceolata* and *P. massoniana* (11.10 and 15.95 mg kg^−1^) (Figure S1a). In contrast, no significant differences were observed in soil available phosphorus (AP) across *C. lanceolata* (0.97 mg kg^−1^)*, L. glaber* (1.33 mg kg^−1^)*, P. massoniana* (1.39 mg kg^−1^), and *S. superba* (1.36 mg kg^−1^). (Figure S1b).

### 2.2. Variation in the Concentration of Foliar P Fractions and Their Relative Allocations

**Figure 2:**
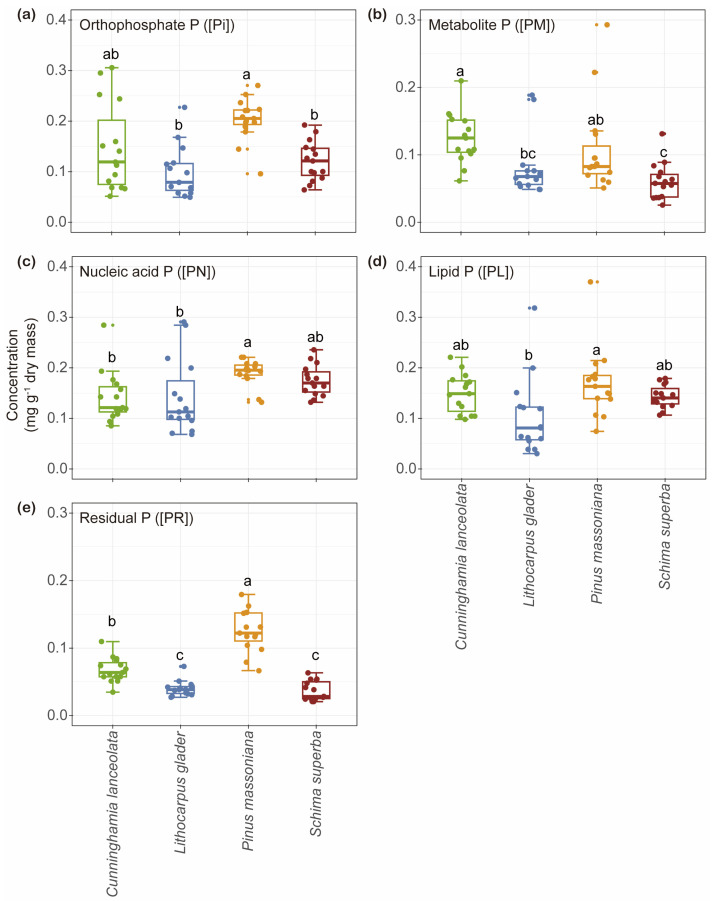
Box plots showing the concentrations of the foliar P fraction (**a**–**e**) among four tree species from the Chinese subtropics. The color of each box pertains to the species. Each point represents an individual tree (*n* = 15). The central box shows the interquartile range and median by species; the whiskers extend 1.5 times the interquartile range or to the most extreme value. Pairwise significant differences among sites are shown as different letters (Tukey’s HSD, *p* < 0.05).

The [Pi] in *P. massoniana* (0.20 mg g^−1^) was significantly higher than in *L. glaber* and *S. superba* (0.10 and 0.12 mg g^−1^) (Figure 2a). Similarly, [PM] in *C. lanceolata* (0.13 mg g^−1^) was significantly higher than in *L. glaber* and *S. superba* (0.08 and 0.06 mg g^−1^) (Figure 2b). Additionally, [PN] in *P. massoniana* (0.19 mg g^−1^) was significantly higher than in *C. lanceolata* (0.14 mg g^−1^) and *L. glaber* (0.14 mg g^−1^) (Figure 2c), while [PL] (0.17 mg g^−1^) was significantly higher than in *L. glaber* (0.10 mg g^−1^) (Figure 2d). Finally, [PR] in both *C. lanceolata* (0.07 mg g^−1^) and *P. massoniana* (0.13 mg g^−1^) was significantly higher than in *L. glaber* (0.04 mg g^−1^) and *S. superba* (0.04 mg g^−1^) (Figure 2e).

**Figure 3:**
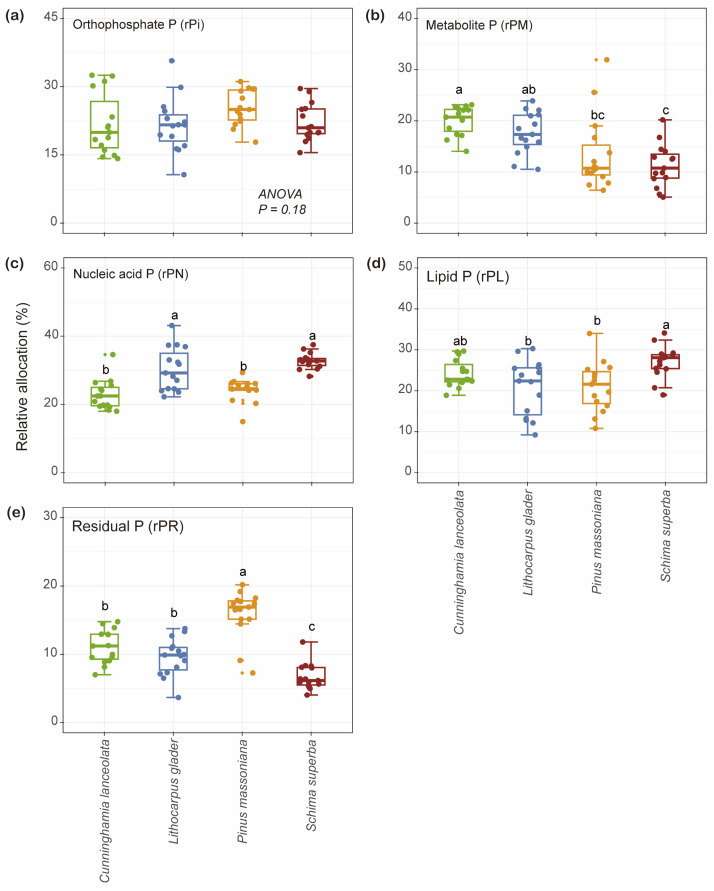
Box plots showing the relative allocations of the foliar P fraction (**a**–**e**) among four tree species from the Chinese subtropics. The color of each box pertains to the species. Each point represents an individual tree (*n* = 15). The central box shows the interquartile range and median by species; the whiskers extend 1.5 times the interquartile range or to the most extreme value. Pairwise significant differences among tree species are shown as different letters (Tukey’s HSD, *p* < 0.05).

No significant differences were observed in the relative allocation of inorganic P in leaf (rPi) among the species (21.73%, 21.62%, 25.38%, and 22.16%, respectively) (Figure 3a). However, *C. lanceolata* (20.08%) showed a significantly higher relative allocation of metabolic P in leaf (rPM) compared to *P. massoniana* and *S. superba* (13.45% and 11.26%), whereas its relative allocation of nucleic P in leaf (rPN) (22.82%) was significantly lower than that observed in *L. glaber* and *S. superba* (30.38% and 32.77%) (*p* < 0.05; Figure 3b,c). In *L. glaber*, rPM (17.64%) and relative allocation of residual P in leaf (rPR) (9.58%) were higher than in *S. superba* (11.26% and 6.70%), but its relative allocation of lipid P in leaf (rPL) (20.79%) was lower (Figure 3b,d,e). Conversely, *P. massoniana* showed relatively lower rPM, rPN, and rPL (13.45%, 24.23%, and 20.99%) among the four tree species but had a significantly higher rPR (15.59%) compared to the others (Figure 3b–e). Meanwhile, *S. superba* showed relatively higher rPN and rPL (32.77% and 27.12%) yet exhibited the lowest rPR (6.70%) (Figure 3b–e).

**Figure 4:**
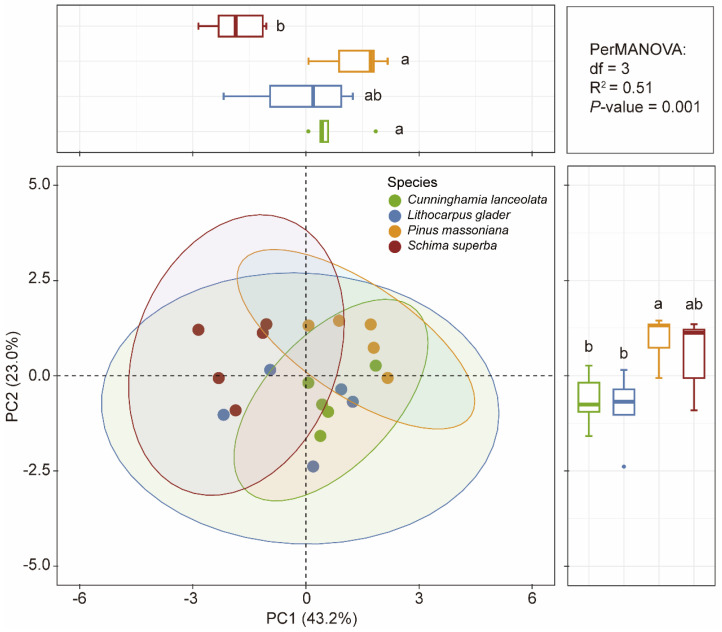
Principal component analysis (PCA) of foliar P fractions among four tree species. Percentages of the total variation explained by the first two PCA axes are given in parentheses. Pairwise significant differences among sites are shown as different letters (*n* = 20).

PCA distinctly differentiated the foliar P fraction compositions among the four studied tree species, as evidenced by the ordination plot (Figure 4). PC1 explained 43.2% of the total variation. While PC2, accounting for 23.0% of the variation, further differentiated *L. glaber* from *P. massoniana* and *S. superba*. Significant differences in foliar P fractions across species habitats were confirmed by a PerMANOVA (df = 3, R^2^ = 0.51, *p* = 0.001). The box plots adjacent to the PCA plot highlight specific group differences in PC1 and PC2 scores, with *P. massoniana* showing significantly different foliar P fraction compositions compared to the other three species (*p* < 0.05).

### 2.3. Correlations Between Leaf P Fractions

**Figure 5:**
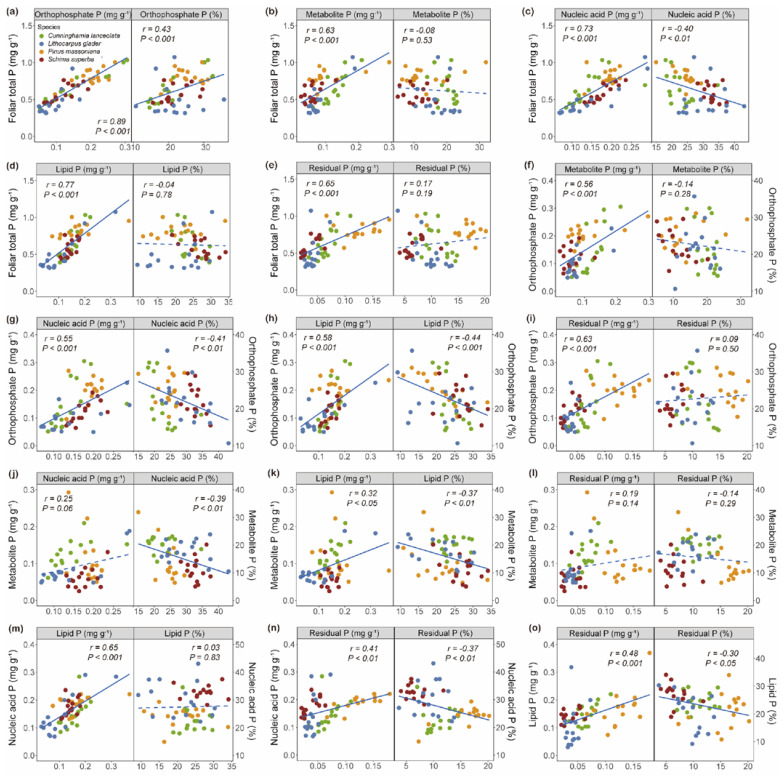
Correlations between concentrations of total P and five leaf P fractions and between the relative allocations of foliar P fractions (five replicates per species, with three individuals in each replicate, (**a**–**e**)). Pairwise correlations between each P fractions, including both concentration and relative allocations (**f**–**o**). Solid and broken lines represent significant and nonsignificant linear correlations, respectively (significant, *p* < 0.05; ns, not significant, i.e., *p* > 0.05; *n* = 60).

The foliar total P concentration was significantly positively correlated with the concentrations of five P fractions—[Pi], [PM], [PN], [PL], and [PR]—across all individuals, among which [Pi] showed the strongest correlation (Figure 5a–e). [Pi] was significantly associated with [PM], [PN], [PL], and [PR] (Figure 5f–i). Notably, [PM] showed a significant positive correlation with [PL] but no correlation with [PN] and [PR] (Figure 5j–l). In contrast, [PN] was positively correlated with both [PL] and [PR], as was [PL] with [PR] (Figure 5m–o).

rPi showed a positive correlation with the foliar total P concentration, whereas rPN demonstrated a significant negative correlation with it (Figure 5a,c). rPi was negatively correlated with those of rPN and rPL (Figure 5g,h). Furthermore, rPM showed significant negative correlations with rPN and rPL (Figure 5j,k). Similarly, the rPN and rPL negatively correlated with rPR (Figure 5n,o).

### 2.4. Linking Leaf Functional Traits and Soil Properties with Relative Allocations of Leaf P Fractions

**Figure 6:**
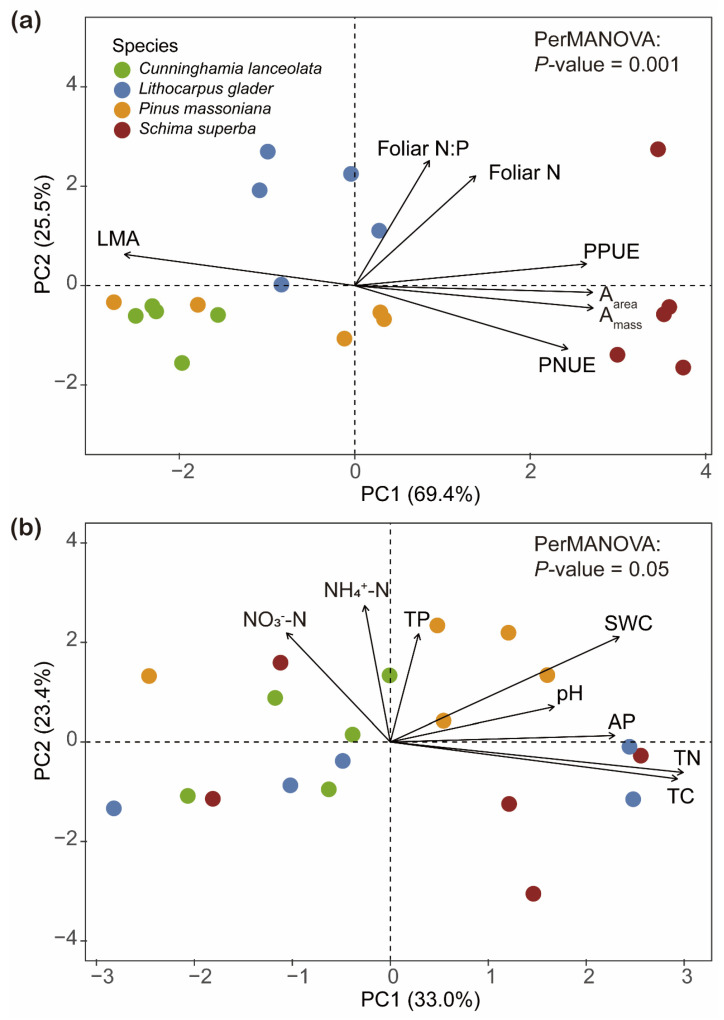
Biplot from principal component analysis (PCA) of variables related to leaf economic traits (LMA: leaf mass per unit leaf area; Foliar N: leaf nitrogen concentration; Foliar P: leaf phosphorus concentration; Foliar N:P: nitrogen to phosphorus ratio; A_area_: area-based maximum photosynthetic carbon assimilation rate; A_mass_: mass-based maximum photosynthetic carbon assimilation rate; PPUE: photosynthetic phosphorus utilization efficiency; PNUE: photosynthetic nitrogen utilization efficiency); (**a**) and soil properties (NO_3_^−^-N: nitrate nitrogen; NH_4_^+^-N: ammonium nitrogen; TP: total phosphorus; AP: available phosphorus; TN: total nitrogen; TC: total carbon; SWC: soil water content); (**b**) across four tree species. Percentages of total variation explained by the first two PCA axes are given in parentheses. Circles represent specific tree species (*n* = 20).

The distinct separation along PC1 highlighted significant differences among these species in resource allocation and nutrient utilization strategies. PC1 accounted for a significant 69.4% of the variance, with LMA, A_mass_, and PPUE showing high factor loadings. Notably, LMA was negatively correlated with both A_mass_ and PPUE. In contrast, PC2 accounted for an additional 25.5% of the variance, identifying leaf [N] and foliar N:P as key contributing variables (Figure 6a). Furthermore, soil properties of PC1 explained 33.0% of the total variance. Meanwhile, soil properties along PC2 accounted for 23.4% of the variance (Figure 6b).

**Figure 7:**
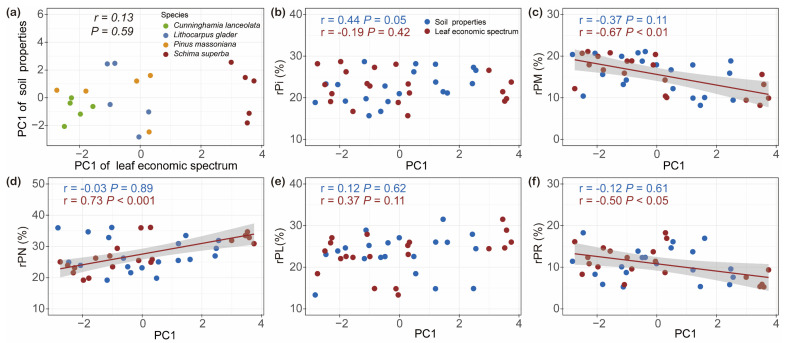
Relationships between species scores along the first axis of principal component analysis (PCA) representing leaf economic spectrum and soil properties (**a**) with relative allocation of inorganic P in leaf (rPi; (**b**)), relative allocation of metabolic P in leaf (rPM; (**c**)), relative allocation of nucleic P in leaf (rPN; (**d**)), relative allocation of lipid P in leaf (rPL; (**e**)), and relative allocation of residual P in leaf (rPR; (**f**)). PC1 of the leaf economic spectrum, calculated by PCA, is a combined index representing interspecific differences in leaf economic traits on LMA, foliar N, foliar P, foliar N:P, A_mass_, PPUE, and PNUE. The higher the value of PC1, the more acquisitive strategies of the tree. PC1, calculated from PCA of soil properties, is a combined index representing interspecific differences in soil properties, including pH, soil water content (SWC), total carbon (TC), total nitrogen (TN), nitrate nitrogen (NO_3_^−^-N), ammonium nitrogen (NH_4_^+^-N), total phosphorus (TP), available phosphorus (AP). An increase in the PC1 value indicates a higher content of bioavailable P in the soil (*n* = 20).

No significant correlation was found between PC1 of the LES and PC1 of soil properties (Figure 7a). However, the analysis revealed distinct correlations between PC1 of the LES and various foliar P fractions across different tree species (Figure 7a–f). Specifically, PC1 of the LES was not significantly correlated with rPi (Figure 7b). In contrast, a strong and significantly negative correlation was identified between PC1 of the LES and rPM (Figure 7c). Furthermore, rPN showed a highly significant positive correlation with PC1 of the LES (Figure 7d). While PC1 of the LES was not significantly correlated with rPL (Figure 7e), it did show a significant negative correlation with rPR (Figure 7f). Importantly, the analysis did not reveal significant relationships between allocations of foliar P fractions and PC1 of soil properties across the studied species (Figure 7a–f).

**Figure 8:**
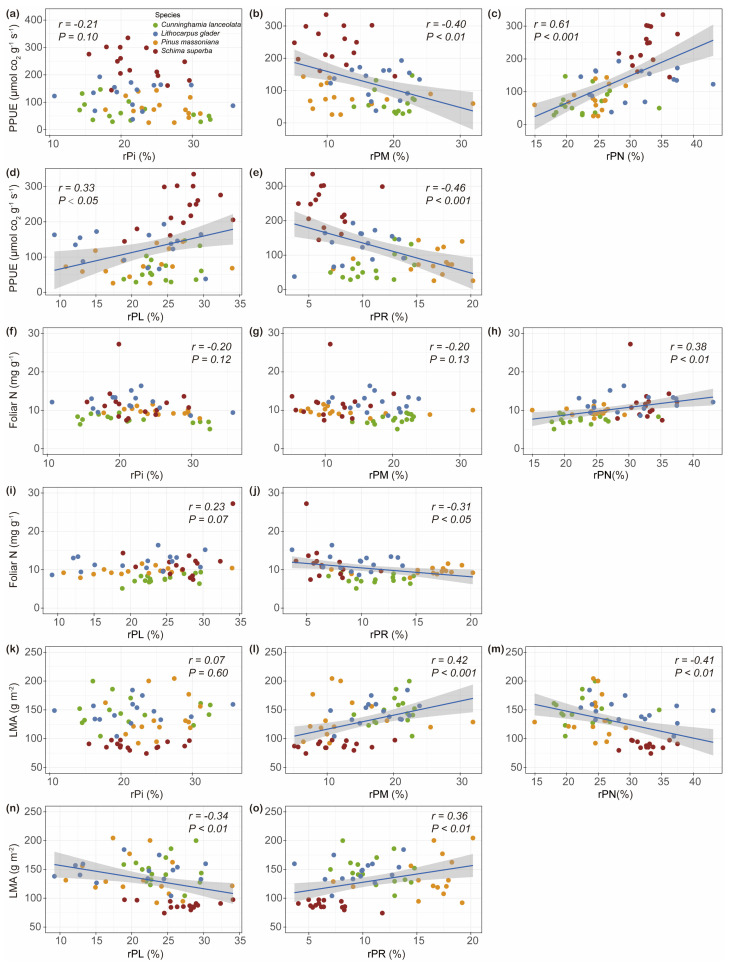
Correlations between allocations of foliar P fractions (rPi: relative allocation of inorganic P in leaf; rPM: relative allocation of metabolic P in leaf; rPN: relative allocation of nucleic P in leaf; rPL: relative allocation of lipid P in leaf; and rPR: relative allocation of residual P in leaf), photosynthetic phosphorus utilization efficiency (PPUE; (**a**–**e**)), leaf nitrogen concentration (foliar N; (**f**–**j**)) and leaf mass per unit leaf area (LMA; (**k**–**o**)) in four subtropical tree species (*n* = 60).

No significant correlation was found between rPi and PPUE (Figure 8a). Notably, rPM was negatively correlated with PPUE (Figure 8b). Conversely, rPN exhibited a strong positive correlation with PPUE (Figure 8c). Additionally, rPL showed a positive correlation with PPUE (Figure 8d), while rPR was negatively correlated with PPUE (Figure 8e). No significant correlations were observed between rPi or rPM and leaf [N] (Figure 8f,g). In contrast, rPN was positively correlated with leaf [N] (Figure 8h). There was no significant correlation between rPL and leaf [N] (Figure 8i), whereas rPR showed a negative correlation (Figure 8j). No significant correlation was found between rPi and LMA (Figure 8k). Notably, rPM was positively correlated with LMA (Figure 8l). Conversely, rPN exhibited a strong negative correlation with LMA (Figure 8m). Additionally, rPL showed a significant negative correlation with LMA (Figure 8n), while rPR was positively correlated with LMA (Figure 8o).

Soil TP was significantly negatively correlated with rPN (Table S2) and positively correlated with rPR. Soil AP was significantly negatively correlated with rPM. In addition, there was a significant positive correlation between NO_3_^−^-N and rPR.

**Figure 9:**
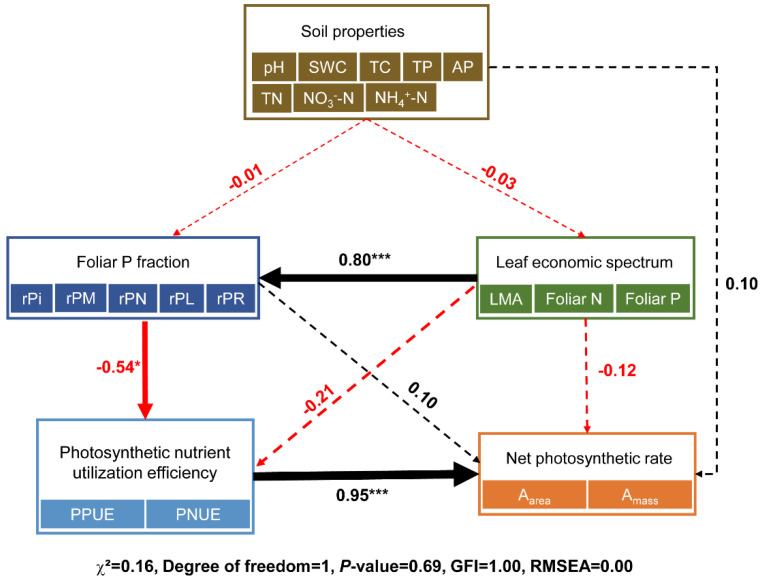
The SEM analysis of the relationships among soil properties (NO_3_^−^-N: nitrate nitrogen; NH_4_^+^-N: ammonium nitrogen; TP: total phosphorus; AP: available phosphorus; TN: total nitrogen; TC: total carbon; SWC: soil water content), foliar P fractions (rPi: relative allocation of inorganic P in leaf; rPM: relative allocation of metabolic P in leaf; rPN: relative allocation of nucleic P in leaf; rPL: relative allocation of lipid P in leaf; and rPR: relative allocation of residual P in leaf), leaf economic spectrum (LMA: leaf mass per unit leaf area; Foliar N: leaf nitrogen concentration; Foliar P: leaf phosphorus concentration), photosynthetic nutrient utilization efficiency (PPUE: photosynthetic phosphorus utilization efficiency; PNUE: photosynthetic nitrogen utilization efficiency), and net photosynthetic rate (A_area_: area-based maximum photosynthetic carbon assimilation rate; A_mass_: mass-based maximum photosynthetic carbon assimilation rate). Black solid lines indicate positive and significant; red solid lines indicate negative and significant; dashed lines indicate non-significant relationships. Multiple-layer rectangles indicate the PC1 from the principal component analysis performed for soil properties, leaf economic traits, foliar P fractions, photosynthetic nutrient utilization efficiency, and net photosynthetic rate. Standardized regression coefficients for each path were given, and results for goodness-of-fit tests were also reported under each plot (*p* > 0.05 indicates a good fit; * *p* < 0.05; *** *p* < 0.001).

The SEM indicated that soil properties did not exert a significant direct effect on foliar P fractions or the LES, nor did they indirectly influence foliar P fractions or photosynthetic nutrient utilization efficiency via the LES (Figure 9). However, foliar P fractions had a significant negative direct impact on photosynthetic nutrient utilization efficiency. Furthermore, a significant positive relationship existed between foliar P fractions and the LES.

## 3. Discussion

Our study uncovered significant interspecific variation in foliar P fraction composition among tree species. However, the strong correlations between PPUE, leaf [N], LMA, and specific P fractions remain evident among four tree species. Crucially, the LES emerged as an influential determinant of foliar P allocation rather than external soil properties. These findings provided valuable insights into the complex interactions among plant physiology, leaf traits, and nutrient allocation, thereby advancing our understanding of plant nutrient economics.

### 3.1. Species-Specific Foliar P Allocation Patterns and Covariations Among P Fractions

Species-specific physiological and ecological adaptations may be key determinants of the P fraction within leaves. Therefore, the relationships between the concentrations and relative allocation of five foliar P fractions were not consistent. Generally, species with rapid growth rates require extensive protein synthesis, corresponding with higher RNA content and thus increased [PN] [1]. Plants tend to exhibit higher photosynthetic rates, necessitating increased metabolic P, which typically leads to a positive correlation between [PM] and [PN] [25]. In our studies, we observed no significant correlation between [PM] and either [PN] or [PR] (Figure 5). This finding contrasts with previous studies that reported strong positive correlations between [PM] and [PN] [7,12]. Indeed, some species may adapt to low-P environments by enhancing protein synthesis efficiency, improving protein stability, and optimizing the use of available P through mechanisms such as increased phosphatase activity [26,27]. This adaptation could lead to an alteration in the expected positive correlation between [PM] and [PN].

A reduced rPi and rPM were strongly associated with an increased rPN (Figure 5). This pattern of trade-offs between investments in [PM] and [PN] has similarly been observed in both subtropical forest species and intraspecific chickpea genotypes [25,28]. Notably, [Pi] and phosphorus-containing metabolites serve as crucial substrates for enzymes involved in photosynthetic processes, such as photophosphorylation and the Calvin–Benson cycle. A reduction in these substrate concentrations may impair enzymatic activities [1,6,29]. In response to reduced substrate availability and to maintain efficient metabolic flux, some plants may enhance their enzymatic investment, which is facilitated by a higher allocation to nucleic acids, predominantly ribosomal RNA (rRNA) [1].

### 3.2. Linkages Between Leaf Functional Traits and Foliar P Fractions

Plants could optimize P allocation among foliar P fractions to achieve high PPUE at the interspecies level. To sustain high PPUE in subtropical trees, reduced rPi and/or rPM may be compensated by an increased abundance of enzymes, as indicated by enhanced investment in nucleic acid P, primarily rRNA. Previous analysis revealed that high-PPUE rice tended to have higher rPN (presumably mainly ribosomal RNA) compared to low-PPUE rice [30].

Reduced rPL through lipid remodeling or substitution with non-phosphorus-containing lipids has been identified as a critical adaptation mechanism to lower overall foliar P demand without sacrificing photosynthetic rates, evident at both species [31,32] and genotype levels [30,33]. However, contrasting these patterns, Wen et al. [25] observed no differences in the concentration and percentage of [PL] among chickpea genotypes with varying PPUE. Our findings suggested a positive correlation between rPL and high PPUE, proposing that high-PPUE species adapt to low-P environments, possibly through increased amounts of endoplasmic reticulum and Golgi apparatus to support rapid ribosomal protein synthesis [34,35]. This maintenance of high phospholipid levels likely involves a strategic reallocation of P resources. Possibly, by concentrating P investment in phospholipids within essential organelles, some species or genotypes optimize organelle function and potentially lower P demands in other cellular structures, thereby enhancing overall P use efficiency [20,25]. This may explain why these species do not significantly reduce their lipid P investment but instead maintain or increase phospholipid levels in specific functional areas, thus ensuring optimal physiological performance. Further investigation into these strategies could deepen our understanding of how plants balance P allocation to maintain high efficiency and adapt to diverse environmental conditions.

There is covariation between [N] and [PN], as rRNA is integral to protein synthesis/turnover, which constitutes the largest fraction of leaf [N] [36]. Our results were consistent with those of various studies that reported positive correlations between [N] and [PN] among diverse species, including 21 Bornean woody species [14], 2 Banksia species [37], and 12 Australian woody species [3]. Nonetheless, our results contradict the findings by Tsujii et al. [12], who observed a positive correlation between [PR] and leaf [N], reflecting [PR]’s role in protein phosphorylation [6]. We found a significant negative correlation, suggesting that [PR] may include other unidentified components that may trade off with phosphorylated proteins. Further research should clarify the relationship between these unidentified components and leaf [N].

### 3.3. Dominant Influence of Leaf Functional Traits over Soil Properties on Leaf Foliar P Fractions

We found that the LES of leaf P fractions is not influenced by soil properties. Notably, a significant negative correlation was observed between rPM and PC1 of LES, while a positive correlation was found between rPN and PC1 (Figure 7). Higher PC1 values, which indicate greater photosynthetic efficiency under low P conditions, were associated with higher photosynthetic phosphorus-use efficiency (PPUE), whereas lower PC1 values correlated with higher LMA. This divergence could be explained by a compensatory mechanism, where reduced substrate concentrations are balanced by increased catalytic efficiency. Specifically, [Pi] and phosphorus-containing metabolites, which serve as substrates in photosynthetic processes like photophosphorylation and the Calvin–Benson cycle, may experience reduced activity due to substrate limitation [1,6,29]. To sustain metabolic flux under these conditions, plants might enhance enzyme efficiency by allocating more resources to nucleic acids, particularly ribosomal RNA (rRNA) [1]. A covariation between PPUE and P allocation to nucleic acids in leaves was observed. In mature leaves, the increased P allocation to nucleic acids, especially rRNA, is crucial for maintaining protein synthesis and turnover [13,25,30]. This includes the continuous production of Rubisco and other enzymes in the Calvin–Benson cycle, as well as the replacement of damaged photosystem proteins, essential for maintaining high photosynthetic rates under low P conditions [38]. Consistent with findings in rice, chickpeas, and ten species of Banksia and Hakea (Proteaceae), which all have rapid growth rates and short lifespans, a positive correlation between rPN and PPUE was observed in mature leaves [13,25,30]. This suggested that enhanced investment in nucleic acids supported protein turnover and photosynthetic efficiency, enabling these species to rapidly replace damaged proteins and adapt to environmental fluctuations through P-enriched ribosomal infrastructure [38,39].

Previous research has demonstrated that soil properties, such as N and P, can influence leaf P fractions [12,15,16,17]. In our study, the positive correlation between soil nitrate-N concentrations and rPR emphasized the critical role of N in facilitating protein synthesis. This was particularly evident in young trees experiencing rapid, year-round growth, where N availability was a key driver of protein synthesis during this vigorous developmental phase [15]. Interestingly, we also observed significant positive correlations between soil TP and rPR, whereas negative correlations were found between soil TP and rPN, as well as between soil AP and rPM (Table S2). Numerous studies have suggested that under conditions of soil P scarcity, plants within species may allocate more P to essential genetic functions, such as nucleic acid P, to support basic cellular processes and adaptability while simultaneously reducing the proportion of P allocated to metabolic activities and more stable pools, such as residual P, in order to prioritize immediate functional needs [16,30,33]. However, SEM analysis offered a broader perspective, revealing that soil properties did not have a significant direct effect on foliar P fractions or the LES, nor did they indirectly influence foliar P fractions through the LES (Figure 9). This outcome may be attributed to the juvenile stage of the trees, which were approximately four years old at the time of the study. At this stage, tree species have a limited influence on soil physicochemical parameters, resulting in minimal variation in soil properties among species. Consequently, the impact of the LES on foliar P fractions appears to be largely independent of soil properties. These findings also suggested that in the early growth phase, inherent traits shaped by the phylogenetic history of tree species may play a dominant role in determining foliar P allocation strategies [2,20,40], potentially outperforming the effects of current soil conditions. Moreover, it is important to note that the limited number of species included in our analysis may restrict the generalizability of our findings. Future studies incorporating a broader range of tree species, encompassing different life histories, mycorrhizal types, and ecological strategies, will be necessary to validate the complex relationships between the LES, soil properties, and foliar P fractions.

## 4. Materials and Methods

### 4.1. Experimental Design

This study was conducted at Baisha Forest Farm in Fujian Province, southeastern China (25°05′ N, 116°42′ E). This area is characterized by a subtropical climate, with an average annual temperature of 19.8 °C and an average annual precipitation of 1637 mm [41]. Before the experimental setup, the site was primarily occupied by a 27-year-old Chinese fir (*Cunninghamia lanceolata* [Lamb.] Hook) plantation. These plantations were clear-cut, and the area underwent slash-and-burn management to prepare for the study. In March 2019, we initiated the experiment by establishing 20 monoculture plots, each 12 m × 12 m. Four species native to the subtropics—*Cunninghamia lanceolata*, *Pinus massoniana, Lithocarpus glaber*, and *Schima superba*—were planted in these plots, with each plot containing 256 systematically spaced trees, approximately 0.75 m apart. To minimize edge effects, a buffer zone of over 2 m was maintained between plots [41]. The experimental design included five replicate plots for each tree species. Within each plot, three trees were randomly selected for analysis.

### 4.2. Photosynthesis Measurement

In July 2023, for each tree, three newly matured branches were chosen from the four cardinal directions at a uniform height. The third and fourth leaves from each branch were used for data collection. Photosynthetic measurements were conducted on three occasions for each leaf to ensure reliability, with the results averaged across trees within each plot. Photosynthesis was quantified using a portable open system (LI-6800; LI-COR; Lincoln, NE, USA). Measurements were performed between 8:30 and 10:30 a.m. on clear, sunny days. A 6 cm^2^ chamber (3 × 2 cm) was employed, where leaf temperature was consistently maintained at 25 ± 0.8 °C and relative humidity of about 70%. The leaves were exposed to a saturating light intensity of 1500 µmol m^−2^ s^−1^ provided by a red-blue light source (6400-02B) and a CO_2_ concentration of 400 µmol mol^–1^. Leaf area for each leaf was determined using Image J software version 1.51j8, which enabled the calculation of both mass-based (A_mass_: maximum photosynthetic carbon assimilation rate per unit leaf mass) and area-based (A_area_: maximum photosynthetic carbon assimilation rate per unit leaf area) photosynthesis rates [31].

### 4.3. Leaf and Soil Sampling

We collected samples of some healthy leaves from multiple branches, including both newly expanded (fully expanded) and older leaves produced in the previous year or earlier. In the field, these leaves were immediately snap-frozen in liquid N_2_ to preserve their biochemical integrity. The samples were then transported to the laboratory, where they were freeze-dried and ground into a fine powder using a wire mill (mesh size 0.08 mm, Retsch ZM200, Haan, Germany).

We systematically collected ten soil cores (each with a diameter of 3 cm and a depth of 0–10 cm) from each of the five plots, transported them to the laboratory, sieved them through a 2 mm mesh, and then air-dried them for subsequent chemical analyses.

### 4.4. Leaf Analyses

Leaf P was sequentially extracted and categorized into five distinct fractions: Pi, PM, PN, PL, and PR, employing the method described by Chapin III and Kedrowski [42], with subsequent modifications by Yan et al. [15]. The Pi fraction was obtained through a modified acetic acid extraction method based on the protocol established by Hurley et al. [43]. Comprehensive descriptions of the P fractionation methodologies are available (Supplementary Materials, Methods S1 and S2). We considered datasets with a recovery rate between 90 and 110%, representing the ratio of the summed concentrations of P in the fractions to the [P_total_]. Leaf [N] was quantified using a Vario MICRO cube elemental analyzer (Elementar Analysensysteme GmbH, Hanau, Germany).

### 4.5. Soil Analyses

Soil water content (SWC) was determined by drying soil samples at 105 °C for 24 h. Soil pH was measured at a 1:2.5 soil-to-water ratio. Soil total carbon (C) and N were analyzed using an elemental analyzer (Elemental EL MAX; Langenselbold, Germany). Ammonium (NH_4_^+^) and nitrate (NO_3_^−^) were extracted from the soil with 1 M KCl at a 1:5 ratio and measured using a Continuous-Flow Auto Analyzer (Bran+Luebbe AA3; Hamburg, Germany). Total soil P was quantified by the molybdenum blue method following digestion with H_2_SO_4_ and HClO_4_ [44,45]. Available P was extracted using 0.5 M NaHCO_3_ at room temperature and measured by the molybdenum blue colorimetric method.

### 4.6. Statistical Analyses

One-way ANOVA, complemented by Tukey’s HSD post hoc test, was utilized to evaluate differences in leaf functional traits, concentrations of foliar P fractions, their relative allocations, and soil properties among various species habitats. Pearson’s correlation tests quantified relationships among leaf P fractions, their relative allocations, and soil properties. Principal component analysis (PCA) was applied separately to the composition of leaf P fractions, leaf economic spectrum, and soil properties to elucidate relationships among tree species. Significant differences among tree species in the ordination space were assessed using PerMANOVA. Additionally, the first and second principal component scores (PC1 and PC2) of leaf P fractions were extracted for each species and analyzed using one-way ANOVA, followed by Tukey’s HSD test, to compare differences. Linear regressions were conducted to determine if PC1 scores from the LES and soil properties were significantly related to the relative allocation of foliar P fractions. A Structural Equation Model (SEM) was employed to examine the potential relationships among leaf functional traits, P fractions, soil properties, photosynthetic nutrient utilization efficiency, and net photosynthesis rate across four tree species. The SEM analysis was performed using the ‘lavaan’ package in R, and the fitting criteria of the structural equations were evaluated based on *p*-values, Chi-square (χ^2^) values, and the Goodness-of-Fit Index (GFI). All statistical analyses were conducted using R software, Version 4.0.2 (R Core Team 2021).

## 5. Conclusions

Our study revealed significant interspecific differences in foliar P fractions and their associations with leaf functional traits and soil properties. Key findings include: (1) Specific foliar P fractions exhibited distinct relationships with leaf functional traits. (2) The allocation of foliar P fractions was more strongly influenced by the leaf functional traits than soil properties. This enhanced understanding is crucial for predicting how tree species adapt to P scarcity in a local ecosystem.

## Data Availability

The datasets generated and/or analyzed during the current study are available from the corresponding author upon reasonable request.

## Supplementary Materials

The following supporting information can be downloaded at: https://www.mdpi.com/article/10.3390/plants14010004/s1, Figure S1: Box plots illustrate soil variables related to soil total P and available P where four species grow in plots. TP: total P; AP: available P. Each box plot is color-coded to represent a different species, with each point on the plot representing an individual tree. The central box of each plot displays the interquartile range and the median value for each species, while the whiskers extend to either 1.5 times the interquartile range or the most extreme data point. Statistical significance of pairwise differences among species is indicated by differing letters, based on Tukey’s Honest Significant Difference (HSD) test at a significance level of *p* < 0.05; Table S1: Soil physiochemical properties in plots where four species grow are presented as mean ± standard error. Identical letters indicate no significant differences between values, based on Tukey’s Honest Significant Difference (HSD) test at a significance level of *p* > 0.05. SWC: soil water content; TN: total nitrogen; TC; total carbon; NO_3_^−^-N: nitrate nitrogen; NH_4_^+^-N: ammonium nitrogen; Table S2: Pearson’s correlation tests between relative allocations to each P fraction and soil properties across four tree species. TP: total phosphorus; AP: available phosphorus; TN: total nitrogen; NO_3_^−^-N: nitrate nitrogen; NH_4_^+^-N: ammonium nitrogen. (See Refs. [14,15,42,43,44,46]).

## Abbreviations

LMA	leaf mass per unit leaf area
A_mass_	mass-based maximum photosynthetic carbon assimilation rate
A_area_	area-based maximum photosynthetic carbon assimilation rate
PPUE	photosynthetic phosphorus utilization efficiency
PNUE	photosynthetic nitrogen utilization efficiency
[Pi]	concentration of orthophosphate P in leaf
[PM]	concentration of metabolic P in leaf
[PL]	concentration of lipid P in leaf
[PN]	concentration of nucleic P in leaf
[PR]	concentration of residual P in leaf
rPi	relative allocation of inorganic P in leaf
rPM	relative allocation of metabolic P in leaf
rPL	relative allocation of lipid P in leaf
rPN	relative allocation of nucleic P in leaf
rPR	relative allocation of residual P in leaf
Leaf [N]	leaf nitrogen concentration
LES	Leaf economic spectrum
