# Supplementary material for: Leaf Phosphorus Fractions Are Coordinated with Leaf Functional Traits in Four Juvenile Tree Species from the Chinese Subtropics
Source: Plants (Basel). 2024 Dec 24;14(1):4. doi: 10.3390/plants14010004 (PMC11722823; doi:10.3390/plants14010004)
Supplement: Supplementary file 1 [file plants-14-00004-s001.zip › plants-3379353-supplementary.pdf]

Supplementary Material

For

Leaf phosphorus fractions are coordinated with the leaf functional traits in four juvenile tree species from the Chinese subtropics

Lei Wang [1†], Jinhong Guan [2†], Zongpei Li [1], Zhijie Chen [3, 4],

Zaipeng Yu [3, 4], Zhichao Xia [1 *]

[1] Anhui Provincial Key Laboratory of Forest Resources and Silviculture, School of Forestry & Landscape Architecture, Anhui Agricultural University, Hefei, 230036, China

[2] College of Life Sciences, Qinghai Normal University, Xining, 810016, China

[3] Key Laboratory for Humid Subtropical Eco-geographical Processes of the Ministry of Education, Institute of Geography, Fujian Normal University, Fuzhou, China

[4] Fujian Provincial Key Laboratory for Subtropical Resources and Environment, School of Geographical Sciences, Fujian Normal University, Fuzhou, China

[†] These authors contribute equally to this work.

[*] Corresponding author: Zhichao Xia, E-mail: zhichaoxia0623@163.com

Methodology for leaf P fractionation analysis：

Phosphorus (P) in leaf tissue was fractionated sequentially into five distinct functional categories: orthophosphate (Pi), metabolite phosphorus (PM), nucleic acid phosphorus (PN), lipid phosphorus (PL), and residual phosphorus (PR), following the established protocols of Chapin and Kedrowski [1], and Kedrowski [2], with subsequent modifications by Hidaka and Kitayama [3], and Yan et al. [4].

Initial Extraction: A 50 mg sample of powdered leaf was weighed into a 2-mL microtube (Tube 1) and extracted with 1 mL of a chloroform-methanol-formic acid mixture (12:6:1 by volume; CMF) for 30-60 seconds. After centrifugation, the supernatant was carefully transferred to a 15-mL tube (Tube 2). This step was repeated three times, using a total of 3 mL CMF.

Second Extraction: The remaining pellet in Tube 1 was subjected to three rounds of extraction with 1.26 mL of a chloroform-methanol-water solution (1:2:0.8 by volume; CMW) for 30-60 seconds each. After centrifugation, the extracts were combined in Tube 2, totaling 3.78 mL CMW.

Phase Separation: To the combined extracts in Tube 2, 1.9 mL of chloroform-washed water was added. The tube was inverted to ensure thorough mixing,

followed by centrifugation to achieve phase separation. This process resulted in a biphasic solution consisting of an aqueous upper layer and a lipid-rich lower layer, separated by a thin, semi-solid interfacial protein layer. The lipid-rich lower layer, containing phospholipids (PL), was carefully transferred to a Kjeldahl flask for acid digestion, while the aqueous layer was transferred to a separate Kjeldahl flask (Tube 3). The residual interfacial layer was rinsed with 1.44 mL of a CMF:CMW:CWW solution (1:1.26:0.62) and subjected to further separation.

Methanol Extraction: The residual pellet in Tube 1 was dried under vacuum and subsequently extracted with 1 mL of 85% methanol for 30 seconds. Following centrifugation, this extract was added to Tube 3.

TCA Extraction: The final pellet in Tube 1 was treated with 1 mL of cold 5% (w/v) trichloroacetic acid (TCA) for one hour, agitating the mixture for one minute every nine minutes. Post-centrifugation, the extract was transferred to Tube 3. This extraction was performed twice.

Composite Fraction and Residual Analysis: The combined extract in Tube 3 represented a mixture of Pi and PM. The remaining pellets in Tube 1 underwent three additional extractions with hot TCA (2.5% w/v) at 95°C for

one hour each. These extractions provided the PN fraction, while the unextractable residue constituted the PR fraction.

Throughout the entire procedure, samples were maintained at 4°C, except during hot-TCA extraction. All extractions employed an Eppendorf Thermomixer set at 1400 rpm. Solid-liquid separations were completed by centrifugation at 1028 × g for 10 minutes at 4°C. Phosphorus concentrations in powdered leaves and each extract were quantified using the molybdenum blue assay [5] via spectrophotometry (UV-mini 1240, Shimadzu Co., Kyoto, Japan), after acid digestion with 3 mL concentrated nitric acid and 1ml concentrated perchloric acid peroxide at 360°C.

Method S2

Pi assay

The concentration of metabolite phosphorus (PM) was determined by subtracting the concentration of orthophosphate (Pi) from the concentration of the composite fraction containing both Pi and PM. The Pi fraction was extracted using an acetic acid method, modified from Hurley et al. [6]. For the extraction, 30 mg of powdered leaf tissue was placed in a 2-mL microtube and mixed with 0.5 mL of 1% acetic acid. The mixture was subjected to oscillation in an oscillator at 1500 cycles per minute for 15 seconds, followed by a 10-second pause; this process was repeated three times. The microtube was then transferred to an ice bath for cooling for 5 minutes, and the oscillation and cooling steps were repeated three additional times. Subsequently, 0.5 mL of fresh 1% acetic acid was added, and the oscillation process was repeated. The sample was then centrifuged at 12,000 RPM for 10 minutes at 4°C, and the supernatant was carefully collected.

The collected supernatant was digested with 3 mL of concentrated nitric acid and 1 mL of concentrated perchloric acid at 360°C. The concentration of Pi in the leaves was then determined using the molybdenum blue method (Murphy

and Riley, 1962) and measured via spectrophotometry (UV-mini 1240, Shimadzu Co., Kyoto, Japan).

References

1. Chapin, III, F.S.; Kedrowski, R.A. Seasonal changes in nitrogen and phosphorus fractions and autumn retranslocation in evergreen and deciduous taiga trees. *Ecology* **1983**, *64*, 376-391. https://doi.org/10.1016/j.foreco.2020.118174
2. Kedrowski, R.A. Extraction and analysis of nitrogen, phosphorus, and carbon fractions in plant material. *J. Plant Nutr.* **1983**, 6, 989-1011. https://doi.org/10.1080/01904168309363161
3. Hidaka, A.; Kitayama, K. Allocation of foliar phosphorus fractions and leaf traits of tropical tree species in response to decreased soil phosphorus availability on Mount Kinabalu, Borneo. *J. Ecol.* **2011,** *99*, 849-857. https://doi.org/10.1111/j.1365-2745.2011.01805.x
4. Yan, L.; Zhang, X.; Han, Z.; Pang, J.; Lambers, H.; Finnegan, P.M. Responses of foliar phosphorus fractions to soil age are diverse along a 2 Myr dune chronosequence. *New Phytol.* **2019**, *223*, 1621-1633. https://doi.org/10.1111/nph.15910

5. Murphy, J.; Riley, J.P. A modified single solution method for the determination of phosphate in natural waters. *Anal. Chim. Acta* **1962**, *27*, 31-36. https://doi.org/10.1016/S0003-2670(00)88444-5
6. Hurley, B.A.; Tran, H.T.; Marty, N.J.; Park, J.; Snedden, W.A.; Mullen, R.T.; Plaxton, W.C. The dual-targeted purple acid phosphatase isozyme AtPAP26 is essential for efficient acclimation of Arabidopsis to nutritional phosphate deprivation. *Plant Physiol.* **2010**, *153*, 1112–1122. https://doi.org/10.1104/pp.110.153270

Table S1 Soil physiochemical properties in plots where four species grow are presented as mean ± standard error. Identical letters indicate no significant differences between values, based on Tukey's Honest Significant Difference (HSD) test at a significance level of P > 0.05. SWC: soil water content; TN: total nitrogen; TC; total carbon; NO_3^--N: nitrate nitrogen; NH_4^+-N: ammonium nitrogen.

Species	pH	SWC(%)	TN (g kg^{-1})	TC (g kg^{-1})	NO_3^--N (mg kg^{-1})	NH_4^+-N (mg kg^{-1})
Cunninghamia lanceolata	4.48±0.50a	29.43±2.86a	1.50±0.84a	27.28±1.17a	2.29±1.08a	21.34±2.18a
Lithocarpus glader	4.51±0.26a	27.30±3.36a	1.64±0.23a	31.56±3.78a	2.74±0.83a	18.19±0.83a
Pinus massoniana	4.66±0.26a	31.94±1.97a	1.50±0.11a	29.94±2.75a	8.05±2.86a	25.76±2.04a
Schima superba	4.60±0.75a	28.64±2.27a	1.62±0.17a	32.16±2.66a	1.57±1.38a	21.44±3.87a

Table S2 Pearson's correlation tests between relative allocations to each P fraction and soil properties across four tree species. TP: total phosphorus; AP: available phosphorus; TN: total nitrogen; NO_3^--N: nitrate nitrogen; NH_4^+-N: ammonium nitrogen.

		TP (mg kg^{-1})	AP (mg kg-1)	TN (g kg^{-1})	NO_3^--N (mg kg^{-1})	NH_4^+-N (mg kg^{-1})
rPi	r	0.14	0.44	0.29	0.02	0.40
	P	0.55	0.05	0.21	0.93	0.08
rPM	r	0.33	-0.54	-0.20	-0.19	0.08
	P	0.15	0.01*	0.39	0.41	0.73
rPN	r	-0.63	0.06	0.00	-0.26	-0.32
	P	0.00**	0.79	1.00	0.26	0.16
rPL	r	-0.19	-0.01	0.14	-0.01	-0.13
	P	0.43	0.97	0.55	0.97	0.57
rPR	r	0.56	0.09	-0.24	0.56	0.11
	P	0.01*	0.70	0.31	0.01*	0.64

Significant correlations are shown with * (P<0.05); ** (P<0.01)

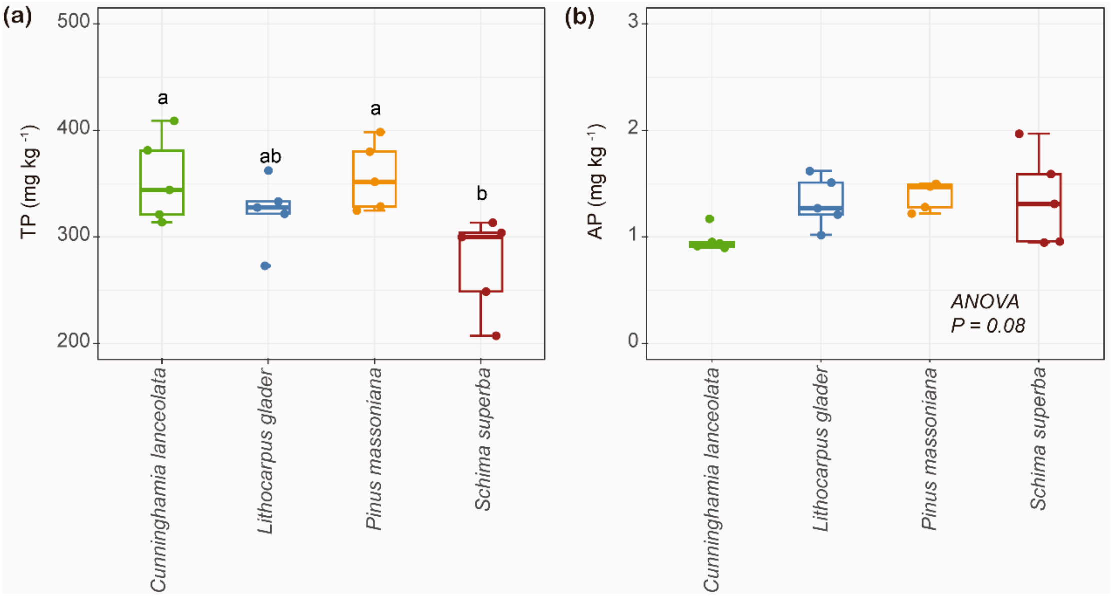

Figure S1 Box plots illustrate soil variables related to soil total P and available P where four species grow in plots. TP: total P; AP: available P. Each box plot is color-coded to represent a different species, with each point on the plot representing an individual tree. The central box of each plot displays the interquartile range and the median value for each species, while the whiskers extend to either 1.5 times the interquartile range or the most extreme data point. Statistical significance of pairwise differences among species is indicated by differing letters, based on Tukey's Honest Significant Difference (HSD) test at a significance level of $P < 0.05$.
